# Design, development and implementation of a national faculty development program to promote CBME in graduate medical education in Switzerland

**DOI:** 10.3205/zma001716

**Published:** 2024-11-15

**Authors:** Andrea Meienberg, Monika Brodmann Maeder, Werner Bauer, Jan Breckwoldt

**Affiliations:** 1 University Hospital Basel and University Basel, Outpatient Medical Department, Basel, Switzerland; 2 Swiss Institute of Medical Education, President, Bern, Switzerland; 3 Swiss Institute of Medical Education, Bern, Switzerland; 4 University Hospital Zurich, Institute of Anesthesiology, Zurich, Switzerland

**Keywords:** faculty development, graduate medical education, clinical teaching, governance

## Abstract

**Background:**

Competency Based Medical Education (CBME) is a global movement in graduate medical training but implementation on a national scale is challenging. One crucial element of fostering CBME is to establish faculty development. We report the design of a national program, the process of implementation, and the results of the first two years.

**Methods:**

Following Kern’s cycle of curriculum development, a group of medical education experts designed a training program covering the basic skills for teaching in clinical settings. In addition, we outlined a qualification pathway for future educators in the program.

**Results:**

The program was built upon 1-day-workshops with the topics: “clinical teaching”, “feedback and assessment”, “clinical leadership”, “supporting trainees in difficulties”. More than 30 workshops were delivered in two language regions to more than 500 clinical teachers. The median rating whether participants’ expectations were met was 9 (of 10 points, IQR 8-9). The qualification pathway for future educators in the program included a nomination, a 2.5-day introductory workshop, shadowing of workshops, and stepwise acquisition of workshop parts as an educator candidate.

**Discussion:**

This faculty development program was well attended and well-received. Using Kern’s established model for the design process including an extensive needs assessment helped to serve the goals of the program. Developing future educators for expanding this program proved resource intensive.

**Conclusion:**

Implementing a national faculty development program was successful based on a rigorous design process, a highly motivated expert team, and learning content tailored to the needs of the audience. Effects on the implementation of CBME still need to be evaluated.

## Zusammenfassung

**Hintergrund:**

Kompetenzbasierte medizinische Weiterbildung (Competency Based Medical Education, CBME) ist derzeit eine weltweite Entwicklung, wobei die Umsetzung auf nationaler Ebene herausfordernd ist. Entscheidende Bedeutung hat dabei die angemessene didaktische Qualifizierung von klinischen Supervisor*innen (Oberärztinnen und Oberärzten). In diesem Beitrag stellen wir ein entsprechendes nationales Programm vor („Teach-the-teacher“-Training), hinsichtlich Konzeption, Umsetzung und Ergebnissen der ersten beiden Jahre.

**Methoden:**

Basierend auf dem Kern'schen Zyklus für Curriculumentwicklung entwicklte eine Expertengruppe ein Traingsprogramm für grundlegende didaktische Kompetenzen in der klinischen Weiterbildung. Ausserdem wurde ein Qualifizierungsprozess für zukünftige Instruktor*innen innerhalb dieses Programms definiert.

**Ergebnisse:**

Das Programm umfasste 1-Tages-Workshops mit den Themen: „Unterrichten im klinischen Alltag“, „Feedback und Assessment“, „Teamarbeit und Leadership“, „Unterstützung von Assisstenzärzt*innen mit Schwierigkeiten“. In zwei Sprachregionen wurden über 30 Workshops für mehr als 500 klinische Weiterbildende abgehalten. Die Teilnehmer*innen bewerteten, „ob ihre Erwartungen erfüllt wurden“, mit im Median 9 Punkten (von maximal 10, IQR 8-9). Der Qualifizierungsprozess für künftige Instuktor*innen im Programm wurde festgelegt über die Stufen der Nominierung, eines 2,5-tägigen Einführungsworkshops, Hospitationen im Progamm und der schrittweisen Übernahme von Workshopteilen als Kandidat*in.

**Diskussion:**

Das „Teach-the-teacher“-Programm wurde gut angenommen. Das Kern’sche Modell erwies sich als geeignetes Verfahren für die Entwicklung. Die Qualifzierung künftiger Instrutor*innen für das Programm war hingegen ressourcenintensiv.

**Schlussfolgerung:**

Wir konnten ein nationales „Teach-the-teacher“-Programm für superviderende Oberärzte und Oberärztinnen erfolgreich etablieren. Erfolgsfaktoren waren ein stringenter Planungsprozesses, zielgruppengerechte Lerninhalte und -ziele, sowie ein hoch motiviertes Expertenteam bei der Entwicklung. Allerdings sind die Effekte auf die Umsetzung von CBME noch offen und müssen zukünftig evaluiert werden.

## 1. Background

To advance training in graduate medical education, Competency Based Medical Education (CBME) has increasingly developed over the past decade. This includes – but is certainly not restricted to – the teaching of the entire scope of the CanMEDS framework [1] and the concept of “Entrustable Professional Activities” (EPAs) [2]. While the CanMEDS framework and EPAs are rather intuitive to understand [3], [4], we still face an immense challenge to translate the concept of CBME into daily practice. For achieving this goal, a bundle of strategies is necessary including accreditation standards, appropriate assessment tools, and effective governance [5], [6]. A further cornerstone for implementing CBME is faculty development on a national scale. It is paramount to support clinical supervisors of the frontline workforce (who generally are non-experts in education) in applying the principles of CMBE. Therefore, training clinical supervisors is a central element of this effort [7], [8].

For this purpose, we designed a faculty development program with a modular structure. A Swiss team of clinical educators (“core faculty”) developed the different workshop units in collaboration with the Royal College of Physicians London (RCP). The project was conceived along the six steps of curriculum development by Kern [9]. This project report gives an overview of the program. In specific, it describes the conception, content and structure of the program, the implementation, and the evaluation of the first two years, as well as the educator development pathway for new course instructors.

## 2. Project description

### 2.1. From problem identification to needs assessment (Kern steps 1+2)

In 2012, the Swiss Institute of Medical Education (SIME) launched a “teach-the-teachers” initiative by inviting the “Royal College of Physicians of London” (RCP) to Switzerland for delivering Teach-the-teacher workshops. Twice a year, educators from the RCP gave a week of workshops in English on the topics clinical teaching, feedback & assessment, and leadership. The interest in these workshops was high and the overall feedback from participants was extremely positive. However, participants suggested having workshops better tailored to the Swiss context including using the languages of the different national regions. In addition, a survey by the SIME among program directors at institutions revealed the need to better anchor CBME principles in the national medical training, thus calling for an expansion of the number and topics of workshops. Further support for the need of a national teacher training was the finding that young clinical supervisors were struggling with the challenges of their new positions including educational competencies [10], [11]. Interestingly, also very experienced clinical supervisors with many years of teaching experience expressed interest in acquiring fundamentals in medical education [8].

As a result, the SIME initiated a process to build a Swiss faculty development program for postgraduate medical education, starting in 2019. This was undertaken as a collaborative process of a working group of Swiss clinical educators, almost all with specific medical education expertise at master’s levels, together with the RCP. This “core faculty” adapted content from the RCP workshops to the Swiss context and added further content relevant to the national needs.

### 2.2. Conception of the program (Kern steps 3 + 4)

#### 2.2.1. Workshops – Topics, goals and objectives, and educational strategies 

Based on the experience and feedback from the RCP Teach-the-teacher courses, empirical data from Switzerland [8], [10], [12], and the rich workplace experience of the expert group, we decided to start with three one-day workshops on the topics “teaching in clinical settings”, “feedback and workplace-based assessment” and “supporting the trainee in difficulties”. The instructional design of each of the workshops was prepared in groups of 3-4 experts and then presented to whole “core faculty” for further refinement. Colleagues from the RCP supported the process as facilitators. Subsequently, when the workshops were delivered for the first time, core faculty members acted as critical observers providing external feedback. The workshops are briefly outlined in table 1 (Tab. 1) regarding content, learning objectives, and target groups. 

All Swiss workshops are held in person. A few days before the workshops, the course documents are sent to the participants by e-mail. Pre-course materials include relevant literature references and a workbook with the key slides of the workshop. 

All workshops used a wide range of didactic strategies including self-reflection, brainstorming, group work, role-play, videos, brief interactive inputs, and plenary discussions. The main instructional approach was team teaching by two to three educators (with two of them being “full educators” (FE) and one “educator candidate” (EC). Further details, see below: “educator development”). The overall educator – participant ratio ranged from 1: 6 to 1:10. To support the national development of CBME, a special focus was put on the concept of Entrustable Professional Activities (EPAs) [2], [4]. Specific reference to EPAs was given in all workshops (“clinical teaching”: the idea of entrustment for future performance; “feedback and assessment”: using entrustment scales in workplace-based assessment, e.g. utilizing mobile technology [13]; “supporting trainees in difficulites”: using entrustment scales to compare to the standard trainee development for creating a common understanding between trainee and supervisor).

In the second year of implementation, the program was expanded by a workshop on “leadership and teamwork” and a 3-day workshop-retreat designed for the target group of young clinical supervisors (summer school). Content wise, the summer school covered all the workshop topics including clinical teaching, feedback & assessment, struggling trainees and basic leadership.

#### 2.2.2. System of workshops: towards a national “teach-the-teacher” program 

**Figure 1:**
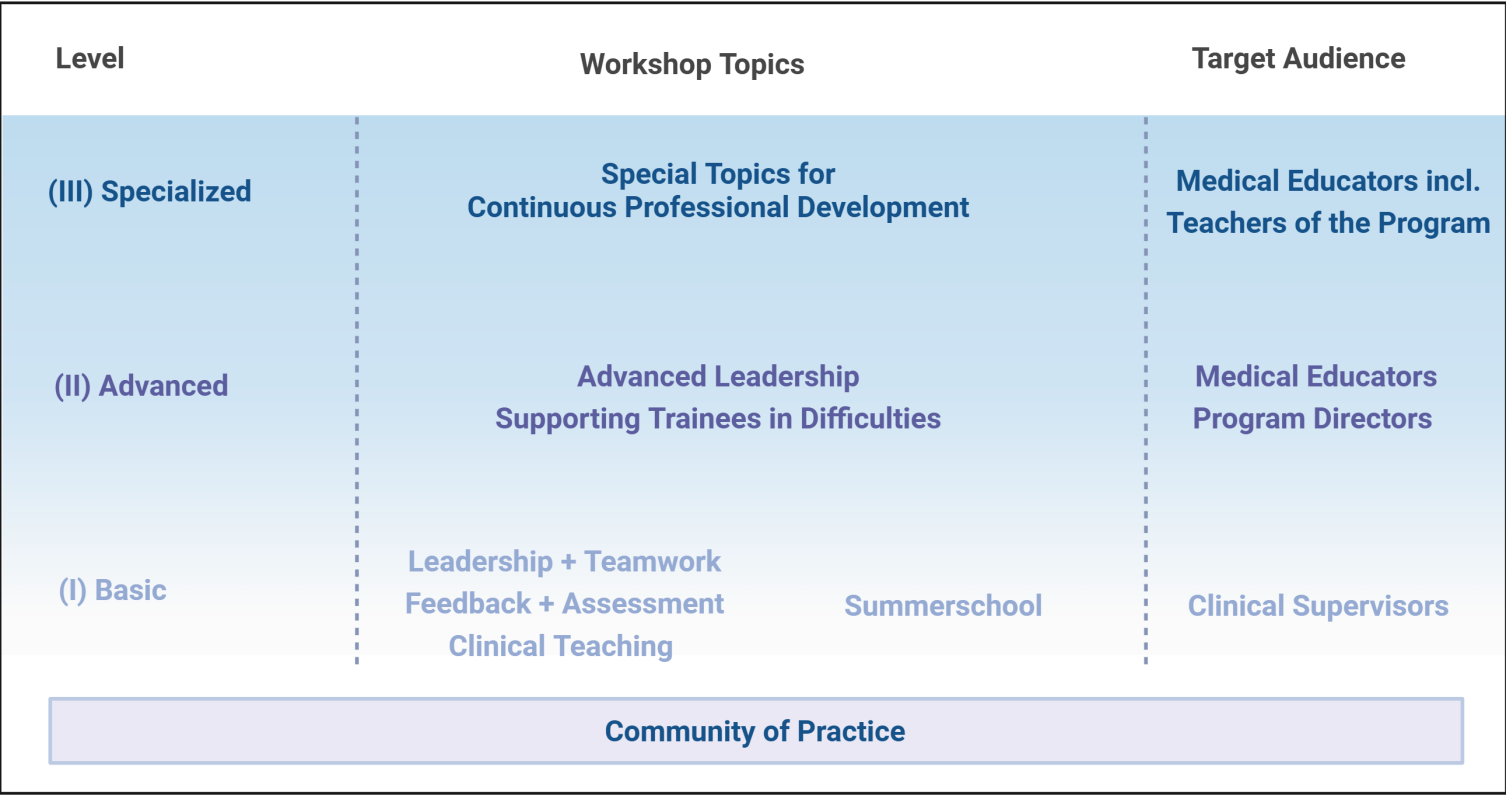
Structure of the SIME Teach-the-teacher program (Created with BioRender.com)

The workshops were fitted into a modular system covering fundamental competencies for clinical teaching in graduate medical education. The program aims to qualify for three levels of educational competencies (see figure 1 (Fig. 1)). 

*The basic level (I) *covers competencies expected from every clinical teacher. The workshops are structured as 1-day modules, which can all be attended separately, and without a specific sequence. As an exception, the Summer School, which also addresses the competencies of the basic level, can only be booked as a 3-day retreat course package.

*The advanced level (II) *addresses the needs of senior educators and program directors at institutions. It is recommended having attended the basic level or equivalent courses beforehand.

*The specialised level (III)* continuously develops skilled faculty members including educators within the “Teach-the-teacher” program to provide opportunities for continuous professional development.

In the individual workshops, cross-references are made to other course modules. For promoting the development towards CBME in the various workplaces, we attached high value to linking participants with each other during the workshops. We pointed at the importance of joining forces with peers in one’s own working environment and of building a community of practice [14]. To ensure the sustainability of the course content of the summer school, an online meeting with participants and instructors was held a few months after the course for exchanging hurdles and obstacles as well as success stories of the implementation of educational activities in the workplace. 

To demonstrate the close link between the SIME program and the RCP, and to maintain the academic dialogue, we felt it important to continue the RCP workshops. Thus, RCP faculty still teaches English workshops (one face-to-face week per year, as well as online workshops). These RCP workshops also fit into the modular system of the program. The SIME courses “clinical teaching”, “feedback & assessment” and “leadership & teamwork” and the “summer school” primarily cover level I. The SIME Workshop “supporting trainees in difficulties” covers level II, while the RCP courses mainly cover levels II and III. As a further link to the RCP, core faculty members have gone through an RCP accreditation process and hold an RCP accreditation.

### 2.3. Implementation (Kern step 5)

For the start, the program included four full-day face-to-face workshops on different topics. The rationale of organizing the program in one-day workshops was to give participants different options to attend, single workshops or in a sequence, according to their needs. Locations for delivery were spread over the different regions of Switzerland, and workshops were offered in French and German (Italian workshops are scheduled for spring 2024). From the outset, the aim was to offer this national program in three of the national languages to better reflect regional framing conditions. Therefore, the core faculty included medical educationalists with the appropriate bi-lingual skills. 

Each workshop lasts from 9:00 (registration) to 16:30 to enable attendance for participants with travelling times of up to 2 hours. From a subjective perspective, the ideal number of participants lies around 22 (+/-4) to provide a learner-friendly atmosphere and ideal group sizes to facilitate small group role-plays, or individual feedback. 

The workshops were held by teams of three educators (for the specific composition, see below, “educator development”). Administrative support is provided by the SIME, and a congress agency. Generally, workshops are delivered to physicians, but non-physicians who fulfil teaching tasks for physicians may participate in selected cases (e.g., clinical psychologists).

On special request workshops can be booked as in-house courses for departments, hospitals, or medical societies. Content wise, these workshops comply with the modular structure of the program, so that topics and learning objectives are equivalent across the program (no “workshop mixtures”). 

The teaching faculty has a mandate by the SIME. To guarantee the financial sustainability of the program, participation fees cover the largest part of the costs. Usually, the participants get their fees refunded by their home institutions. However, to fully support the program during the implementation phase, the SIME covered the financial balance.

By the end of 2023, more than 50 one-day workshops and three Summer Schools have been delivered in two language regions to more than 600 clinical supervisors. 

### 2.4. Evaluation and feedback (Kern step 6) 

#### 2.4.1. Evaluation process

Each workshop is evaluated by the participants with a written evaluation form. The questionnaire is kept simple in order not to overburden participants and educators and to ensure authentic feedback. Participants are asked whether their expectations were met by the workshop, whether the content was relevant to their daily clinical work, what they benefited from most and what should be improved in the future. For rating whether expectations had been met and of the relevance for daily clinical work, Likert-like scales were used ranging from 0 (expectations not being met at all) to 10 (expectations absolutely fulfilled) and from 0 (not at all useful in daily work) to 10 (very easy to implement). Semi-open questions were used to ascertain which workshop parts were considered particularly valuable and to provide the chance of making specific suggestions for improvement. Such suggestions were made in respect to, e.g., the ratio between plenary and small group phases, or the usefulness of certain teaching tools. 

Furthermore, after each workshop the faculty conducts a debriefing. This includes a structured workshop report with basic information from the perspective of the faculty as well as a summary of the development of educator candidates (further details, see below). In selected cases (e.g., quality management, development of new workshop), course observations are held by medical education experts using a more detailed observation form. 

The group of Swiss educators who have been developing the workshops from the beginning (in close collaboration with the RCP) had an important role in continuously evaluating and further developing the program. A regular newsletter was distributed to keep all group members updated and an annual retreat was held for program evaluation, planning and further development. The meetings also support the community of practice among the workshop facilitators and are used for professional inputs for the continuous training of the teaching faculty. 

#### 2.4.2 Results of evaluations by participants 

In the first two years, around 30 workshops were delivered by the Swiss faculty. The overall evaluations are shown in table 2 (Tab. 2). For the first workshops, the median rating whether the participants’ expectations had been met was 9 (out of 10; IQR 8-9), and the transferability to daily clinical practice was rated with a median of 8.5 (IQR 8-9).

### 2.5. Sustainability and expansion: Educator development 

Clinical credibility was regarded central for the acceptance of the program by workshop participants. Therefore, it was a fundamental principle that all faculty members were well founded in clinical supervision routine. To be capable of offering a further expanding program, it was necessary to broaden the educator group. Hence, the acquisition of new workshop teachers began at an early stage. 

We related educator eligibility to a set of qualifying aspects including a strong commitment in education, the solid knowledge of RCP/SIME facilitated courses, a statement of commitment for the program, and educational expertise already acquired (such as, but not restricted to, medical education master programs). Educator development goes through a stepwise process with the stages: “Educator Potential” (EP), “Educator Candidate” (EC), “Full Educator” (FE). After nomination, future educators (with EP) are introduced to the program by a 2.5-day introductory workshop. Delivered by RCP educators and “core faculty” members, this workshop provides an in-depth familiarization with the SIME “teach-the-teachers” course content, and special lessons on effective teaching skills.

Thereafter, candidates (as ECs) participate as shadowing observers in 1-2 workshops of one topic. Thereafter, ECs assist the two FEs of the workshop in teaching at least one relevant section of the workshop under supervision of the FEs present. The workshop debriefing includes structured feedback for the ECs followed by a mutual agreement on the next developmental steps. Based on evaluation meetings of the core faculty twice a year, it is determined when an EC is promoted to be a full educator. Until the end of 2023, around 5 persons have been promoted to FEs status, and a further 20 are at different EC stages.

## 3. Discussion

Triggered by the intention to fundamentally anchor CBME in graduate medical education in Switzerland over the next 10 years [6] the demand for training clinical supervisors is rapidly increasing. As a result, a modular “teach-the-teacher” program with three different levels of competence was established and a core faculty of clinical educators was built up.

### 3.1. Process of designing the program

The development from idea to implementation took around three years. An important factor of success was the structured team approach of designing the initial set of workshops, first in small expert groups, then by discussing all workshops in the core faculty and finally observing the first deliveries. Thereby, redundancies between workshops could be reduced, a consistency over the program could be established and an ownership for all “core faculty” was reached. Such iterative processes have been proven successful in other educational contexts [15]. A further factor of success was the presence of RCP colleagues who ensured some continuity between the “original” RCP workshops and the Swiss program. 

During the development process, about half of the members of the original group left the project before the program had started. Several reasons can be identified for this. First, the development of the program was time-consuming and required a strong commitment from the faculty members as well as from their institutions which had to make time available to their staff. Second, continuity was important, and it was not possible for some of the members to keep up the commitment while also working in full clinical practice. However, the whole process of developing the program has brought the rest of the core group together and increased their enthusiasm for the project, thus establishing elements of a community of practice [14].

### 3.2. Attendance of workshops: factors of success

The workshops were well attended right from the start. This success was primarily due to the strong support of the SIME governance and the national need for implementing CBME including EPAs. Implementation of such programs is highly dependent on reliable institutional and financial support [5], while it is also important to strive towards self-sustainability within the project on the long run. As a second success factor, the workshops were advertised on the SIME website and through personalized emails to all Swiss program directors for graduate medical training. However, as the announcements were not always forwarded to the target group of young clinical supervisors, adjustments to the advertising channels will have to be made.

A third factor of success may have been the program’s close connection to the well-known and well-received RCP workshops. In the legacy of these workshops and as an official project of the SIME, the credibility within Switzerland was likely to be high. As the final success factor it can be assumed, that the positive evaluations of the workshops have led to positive personal recommendations of the workshops. 

### 3.3. Was the target group reached?

In general, the workshop announcements addressed participants across all clinical disciplines. In consequence, all workshops were interdisciplinary by participants, which enriched the personal exchange of experiences. However, this also posed the challenge that not everyone felt equally comfortable with e.g., the role-play examples, or the teaching tools presented. 

To enable participants to transfer the course content to their own working environment, we provided a highly interactive and reflective setting, explicitly discussing after each thematic block how to incorporate the learning content into practice in the specific working environment of the home institution. Based on this feedback, we continuously adapted the workshop content so that all disciplines felt seen in the examples. A further strategy to better reach the broad spectrum of participants was to ensure that the teaching faculty was an interdisciplinary and gender-mixed team. 

### 3.4. Educator development

After the program was successfully launched, one of the biggest challenges was to recruit qualified new teachers for the project. When it comes to teaching in workshops, facilitators are under scrutiny. Quality criteria had to be defined, and an educator development pathway devised that would account for the different preexistent levels of training. Established educator pathways from other medical fields, such as from resuscitation training [16], served as a model. A challenge of the program expansion is the relatively large number of facilitators to be introduced into the workshops. Feeding new candidates into the program needs a subtle balance between candidates being thrown into teaching too early vs. waiting too long for training opportunities. To evaluate whether educator development leads to the desired results, still needs to be studied [17], [18].

### 3.5. Outlook

To further strengthen the role of teaching skills in medicine in Switzerland, the SIME took an initiative to align faculty development approaches across the country. A working group of representatives from undergraduate and postgraduate medical education is developing criteria for a national Swiss quality label for medical educators. It aims at a nationwide comparability of educator qualifications with special attention to CBME principles. 

## 4. Conclusions

Implementing a national faculty development program was successful. Success factors can be summarized as:

- the design process followed a rigorous and well-established model,
- a highly motivated expert team persued until achieving success,
- the learning content and educational strategies were sufficiently tailored to the needs of the participants,
- full governance support was provided by the Swiss Institute for Medical Education,
- investment in developing future educators was made at an early stage.

However, the effects of the program on the implementation of CBME in Switzerland still need to be evaluated.

## Notes

### Abbreviations

- CBME: Competency-Based Medical Education
- EC: educator candidate
- FE: full educator
- EP: educator potential
- EPA: Entrustable Professional Activity
- RCP: Royal College of Physicians (of London)
- SIME: Swiss Institute for Medical Education (SIWF/ISFM)

### Availability of data and materials

The datasets used and/or analyzed during the current study are available from the corresponding author on reasonable request, or are included in the supplementary information files of this article.

### Authors’ contributions

AM and JB conducted the data collection and analysis and wrote the manuscript in close collaboration. MBM and WB made important intellectual contributions during the writing process, and provided oversight to the whole project. All authors read and approved the final version. 

### Authors’ information

AM is a consultant at the Outpatient Medical Department at the University Hospital Basel and Co-chair of the Teach-the-Teacher program of the SIME. She is specialized in internal medicine and has a Master in Medical Education from the University of Bern. 

MBM is President of the Swiss Institute of Medical Education. She is specialized in emergency medicine, internal medicine and has a Master in Medical Education from the Universities of Bern and Chicago.

WB is Past-President of the Swiss Institute of Medical Education and was specialized in internal medicine and oncology. 

JB is consultant at the Institute of Anesthesiology, University Hospital Zurich, Zurich, Switzerland, co-chair of the Teach-the-Teacher program of the SIWF/ISFM, and member of the EPA committee of the SIWF/ISFM.

### Authors’ ORCIDs

- Andrea Meienberg: [0000-0002-0751-2469]
- Monika Brodmann Maeder: [0000-0001-5608-7887]
- Jan Breckwoldt: [0000-0003-1716-1970]

## Acknowledgements

Core Faculty for designing and further developing the specific workshops: Kurt Albermann, Maya Bose, Tiziano Cassina, Stefan Eisoldt, Regula Fankhauser, Sonia Frick, Priska Grünig, Henry Madlon, Marco Manchietti, Christopher Richard. 

RCP for supporting the development: David Perry and Tom Baker on behalf of the entire RCP team.

AM, MBM and JB want to express their deepest gratitude to Werner Bauer. Werner who recently deceased after prolonged severe disease primarily initiated the Swiss teach-the-teacher program. He based his initiative on the strong belief that teaching institutions also provide superior patient care. Without him, the program would never have come to life

## Competing interests

AM is co-chair of the SIME programme, MBM is the president of the SIME, WB is past president of the SIME, JB is Co-chair of the SIME Teach-the-teacher programme.

## Figures and Tables

**Table 1:**
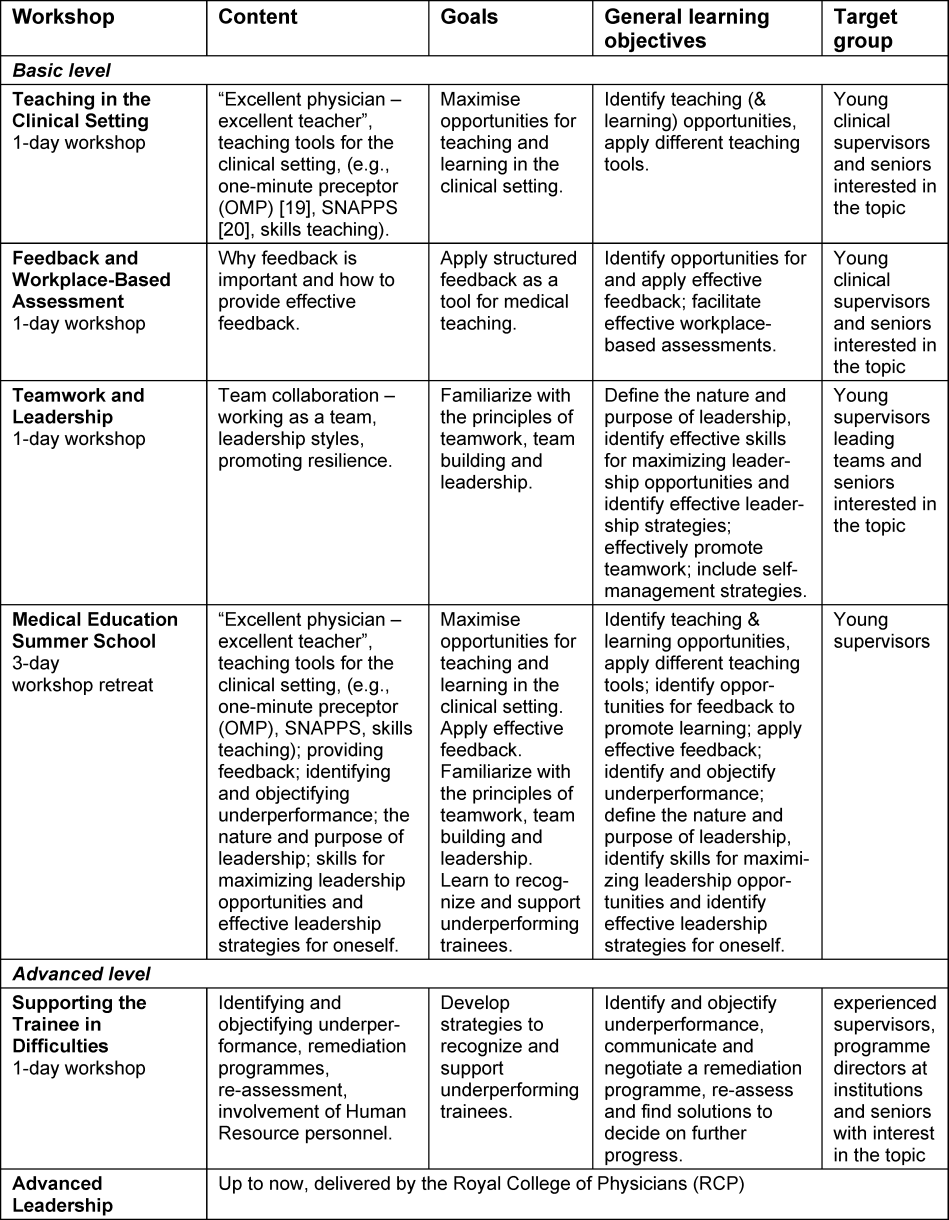
Overview of workshop content, goals, learning objectives, and target groups

**Table 2:**
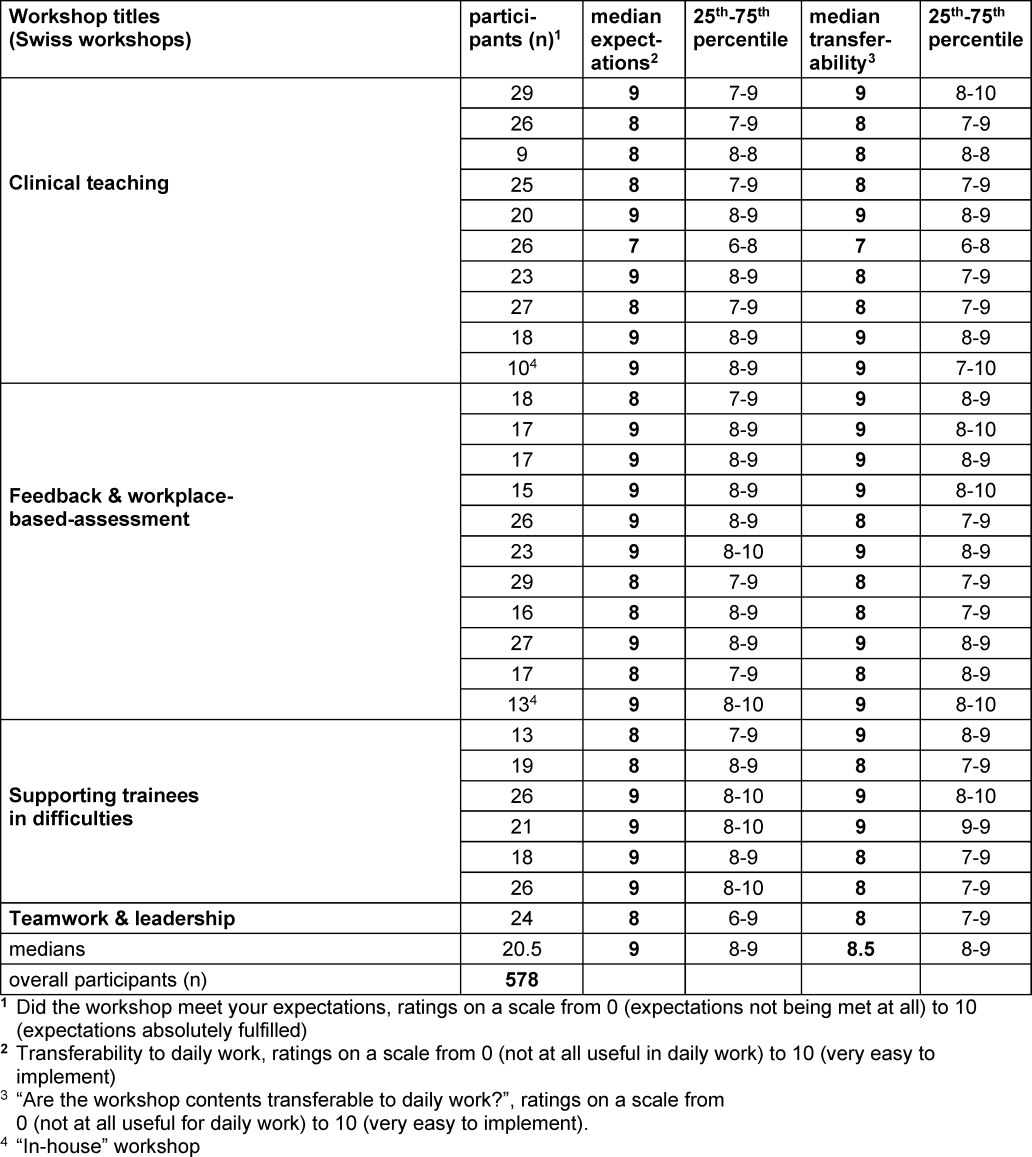
Overall evaluation by participants (Swiss workshops)
